# Translation of Chinese version of the measure of audiologic rehabilitation self-efficacy for hearing aids and the self-efficacy among hearing aid users in China: Application of the questionnaire

**DOI:** 10.1371/journal.pone.0330163

**Published:** 2025-08-20

**Authors:** Jiamei Chen, Ying Shen, Quanran Lin, Yanmei Feng, Hui Li

**Affiliations:** 1 Department of Otolaryngology–Head and Neck Surgery, Shanghai Jiao Tong University Affiliated Sixth People’s Hospital, Shanghai, China; 2 Shanghai Key Laboratory of Sleep Disordered Breathing, Shanghai, China; 3 Department of Otolaryngology, Shanghai Tenth People’s Hospital, School of Medicine, Tongji University, Shanghai, China; 4 School of Medical Technology and Information Engineering, Zhejiang Chinese Medical University, Hangzhou, Zhejiang, China; 5 Department of Ophthalmology, Shanghai Jiao Tong University Affiliated Sixth People’s Hospital, Shanghai, China; Sri Ramachandra Institute of Higher Education and Research (Deemed to be University), INDIA

## Abstract

**Objectives:**

To evaluate reliability and validity of the Chinese version of the Measure of Audiologic Rehabilitation Self-Efficacy for Hearing Aids (C-MARS-HA) and to analyze self-efficacy of Chinese hearing aid users.

**Design:**

The Brislin translation model was strictly adopted to modify the C-MARS-HA. Participants included 134 hearing aid users aged 20–98. Hearing aid users were surveyed through questionnaire interviews regarding self-efficacy and overall satisfaction with hearing aids. Reliability and validity of the questionnaires were analyzed. Descriptive statistical and correlation analyses were conducted on the total score and four factors of the C-MARS-HA and overall satisfaction.

**Results:**

In reliability analysis, Cronbach’ s α, Spearman-Brown, and Guttman split-half coefficients of the overall and four factors were all greater than 0.6, indicating that C-MARS-HA has acceptable reliability. In validity analysis, C-MARS-HA consisted of 24 items across 4 factors (basic handling, aided listening, adjustment and advanced handling) and had acceptable content validity. Exploratory factor analysis yielded with a four-factor solution, explaining more than 40% of the total variance and the loading coefficient for each of the 24 items was above 0.4, indicating that C-MARS-HA had good construct validity.

Average scores for basic handling and adjustment were at least 80%, whereas average scores for aided listening, advanced handling, and total scores were less than 80%. A total of 65.67% of users had an overall satisfaction rate of 80%. A significant positive correlation (p < 0.001) was found between the total score of the questionnaire, the scores of the four factors, and overall satisfaction.

**Conclusions:**

The reliability and validity of the C-MARS-HA is acceptable. This questionnaire can be used for clinical evaluation of self-efficacy among Chinese hearing aid users.

## Introduction

Hearing aids are commonly used for hearing-impairment rehabilitation. Factors that impact hearing aid effectiveness can be categorized as audiological and non-audiological. Non-audiological factors (self-efficacy and satisfaction with hearing aids) also have a significant impact on individuals with hearing deficits initiating and continuing to use hearing aids [1–3]. The effectiveness of hearing aids can be evaluated using subjective and objective methods. Subjective evaluation frequently involves users completing a questionnaire to understand their perceptions of the hearing aid effectiveness in their daily lives [4].

Research has confirmed that self-efficacy plays a positive role in disease treatment and rehabilitation, such as for tinnitus [5], diabetes [6], and other diseases. The self-efficacy of hearing aid users refers to their level of confidence in their ability to use hearing aids, reflecting their ability to adapt to and respond to changes brought about by hearing aids. Self-efficacy has a significant impact on the sustained use of hearing aids [7]. Users with high self-efficacy are more inclined to initiate and continue the use of hearing aids to cope with hearing difficulties. In contrast, users with low self-efficacy may limit or abandon their use of hearing aids [8]. Adherence to hearing aids is associated with improving listening ability, reducing activity restrictions caused by hearing impairment, improving cognition, and preventing falls [8–11].

The Measure of Audiologic Self-Efficacy for Hearing Aids (MARS-HA) questionnaire was developed by West and Smith to assess the level of confidence of hearing aid users in their ability to use and maintain hearing aids in diverse listening environments [7]. The MARS-HA consists of four factors. Factor 1 is aided listening (such as listening in quiet or noisy environments). Factor 2 is basic handling (such as battery installation). Factor 3 is adjustment (such as adapting to sounds heard after wearing the hearing aid), and Factor 4 is advanced handling (such as identifying different components of the hearing aid). These four factors are the main factors affecting the self-efficacy of hearing aid users. Experts have suggested that the self-efficacy score of hearing aid users should reach 80% [8]. Audiologists can determine the level of confidence of hearing aid users in aided listening, basic handling, adjustment and advanced handling with the MARS-HA. Audiologists can record the specific reasons why users feel lacking in confidence through further conversations with users. Therefore, the MARS-HA can estimate a new user’s ability to use hearing aids and analyze the difficulties of experienced users to assist audiologists in providing targeted guidance and suggestions. Evaluating and comparing the results of the MARS-HA before and after an intervention can reveal the effectiveness of the rehabilitation treatment [7,8,12]. The MARS-HA currently has versions in English [7,13–17], Spanish [18], and French [19], all of which have acceptable reliability and validity.

The ultimate goal of conducting the MARS-HA questionnaire evaluation for experienced hearing aid users is to identify the difficulties they face when using hearing aids, provide rehabilitation advice, improve the effectiveness of the hearing aids, and help improve adherence and success in using hearing aids. Considering the positive effects and widespread applications of the self-efficacy and MARS-HA as elaborated above, the first aim of this study was to translate the MARS-HA into a Chinese version and determine/verify its reliability and validity. The second aim of this study was to analyze the self-efficacy of hearing aid users in China based on the results of the C-MARS-HA survey, thereby providing a reference for clinical applications.

## Materials and methods

### Participants

All participants were recruited at the Sixth People’s Hospital of Shanghai between June 1 and June 30, 2024. Selection criteria included: ① Age ≥ 18 years; ② using hearing aids in one or both ears for at least 6 months; ③ no obvious language, speech, or cognitive barriers.

### Ethical information and informed consent

The ethics had been approved by the Ethics Committee of Shanghai Sixth People’s Hospital (Approval No. 2023−176). All participants read and signed a written informed consent form personally or with the assistance of their families.

### Translation of Chinese version of the MARS-HA questionnaire

**Fig 1:**
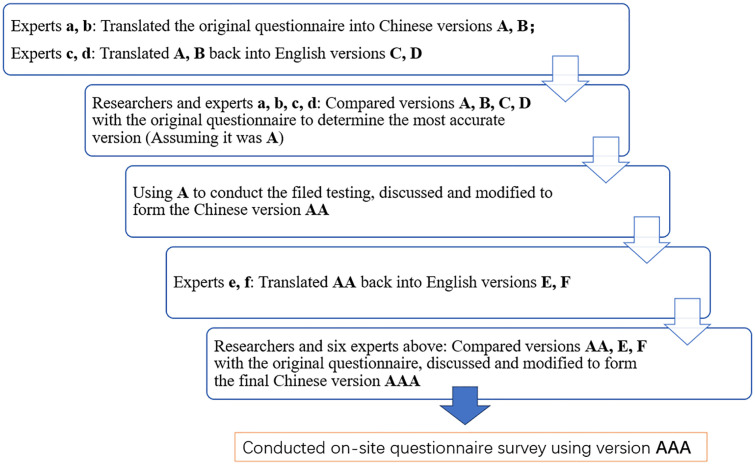
The translation process of the questionnaire.

The adjusted version of the Brislin translation model proposed by Jones was strictly adopted for the translation [20]. Researchers and six experts participated in the translation of the questionnaire. All six experts are otolaryngologists or audiologists with professional backgrounds in otology and audiology. The translation process of the questionnaire is shown in Fig 1.

First, two audiologists from different dialect regions proficient in English and Chinese, respectively translated the English version of the MARS-HA into Chinese. Second, two other bilingual experts translated the two versions of the translated questionnaire back into English. Third, the researchers collaborated with the four aforementioned experts to compare the four translated versions with the original English versions. The most accurate and easily understandable Chinese version was determined.

The selected Chinese version questionnaire above was used for file testing. a small sample of 30 participants participated in the test. Tested the comprehensibility, logicality, and cultural adaptability of this questionnaire. Participants were required to independently complete the questionnaire, record and provide feedback on whether there were any logical errors or reading difficulties during the filling process. Immediately provided feedback on the above situation to the researchers after completing the test. Based on feedback, researchers and experts communicate and adjust Chinese expressions appropriately to better achieve cultural adjustment.

Two other bilingual experts then translated the Chinese version into English. Subsequently, all researchers and the six experts jointly discussed and compared the translated Chinese and English versions of the questionnaire with the original English version, ultimately achieving consistency between the Chinese and English versions in terms of concepts, semantics, and context. Cultural adjustments were included to make the questionnaire as understandable as possible. Finally, a Chinese version of the Measure of Audiologic Rehabilitation Self-Efficacy for Hearing Aids(C-MARS-HA) was developed.

Familiarity check was also conducted during the questionnaire translation process. The questionnaire was developed after a familiarity check from Chinese language experts then it was intended for content validation by 6 experts with professional backgrounds in otology and audiology.

### Tool and processes

The C-MARS-HA tool consists of 24 items divided into four factors: aided listening (items 16–24), basic handling (items 1–5, 7, and 10), adjustment (items 13–15), and advanced handling (items 6, 8, 9, 11, and 12) [7]. The Chinese and English versions of the 24 items are listed in Table 1. A column of 0% −100% numbers (0%, 10%, 20%, until 100%) is located below each item, wherein 0% represents “no certainty in one’s capability” and 100% represents “complete certainty.”

**Table 1:** Items of the MARS-HA and results of exploratory factor analysis.

Items (English and Chinese versions)	Factor load coefficient				Commonality
	Factor 1	Factor 2	Factor 3	Factor 4	
1. I can insert a battery into a hearing aid with ease. 我可以很容易地把电池装到助听器里。	0.112	**0.807**	0.034	0.207	0.708
2. I can remove a battery from a hearing aid with ease. 我可以很容易地把电池从助听器里取出。	0.153	**0.877**	0.116	0.137	0.824
3. I can tell a right hearing aid from a left hearing aid. 我能分辨出助听器是左耳的还是右耳的。	0.009	**0.884**	0.129	0.058	0.802
4. I can insert hearing aids into my ears accurately. 我可以准确地把助听器放入我的耳朵。	0.091	**0.905**	0.193	0.103	0.876
5. I can remove hearing aids from my ears with ease. 我可以很容易地把助听器从耳朵里拿下来。	0.052	**0.908**	0.145	0.102	0.859
6. I can identify the different components of a particular hearing aid (i.e., microphone, battery door, vent, etc.). 我可以识别特定助听器的不同部件 (比如麦克风、电池仓、通气孔等)。	0.234	0.177	0.153	**0.731**	0.644
7. I can operate all the controls on a particular hearing aid (knobs, switches, and/or remote control) appropriately. 我可以操作助听器上的所有按钮(旋钮、开关、遥控器 (如有)等)。	0.050	**0.572**	−0.070	0.299	0.424
8. I can stop a hearing aid from squealing. 我可以使助听器不发出啸叫声。	0.211	0.156	0.123	**0.786**	0.702
9. I can troubleshoot a hearing aid when it stops working. 助听器有问题的时候, 我可以进行故障排除。	0.287	0.158	0.071	**0.802**	0.756
10. I can clean and care for a hearing aid regularly. 我会定期清洁和保养助听器。	0.096	**0.510**	0.187	0.352	0.428
11. I can name the make or model of a particular hearing aid. 我可以说出助听器的牌子或型号。	0.024	0.108	0.136	**0.770**	0.624
12. I can name the battery size needed for a specific hearing aid. 我可以说出助听器所需的电池型号。	−0.032	0.207	0.210	**0.658**	0.521
13. I could get used to the sound quality of hearing aids. 我可以习惯助听器的音质。	0.245	0.176	**0.883**	0.237	0.927
14. I could get used to how a hearing aid feels in my ear. 我可以习惯助听器在我耳朵里的感觉。	0.181	0.193	**0.892**	0.259	0.932
15. I could get used to the sound of my own voice if I wore hearing aids. 如果我戴上助听器, 我可以习惯我自己的声音。	0.237	0.230	**0.889**	0.209	0.944
16. I could understand a one-on-one conversation in a quiet place if I wore hearing aids. 如果我戴上助听器, 我可以在安静的地方听懂一对一的谈话。	**0.617**	0.238	**0.466**	0.087	0.662
17. I could understand conversation in a small group in a quiet place if I wore hearing aids. 如果我戴上助听器, 我可以在安静的地方听懂一对多人的谈话。	**0.766**	0.029	0.215	0.074	0.640
18. I could understand conversation on a standard telephone if I wore hearing aids. 如果我戴上助听器, 我能通过电话进行交流。	**0.751**	0.124	0.195	0.103	0.627
19. I could understand television if I wore hearing aids. 如果我戴上助听器, 我能看电视。	**0.776**	0.094	0.055	0.012	0.614
20. I could understand the speaker/lecturer at a meeting or presentation if I wore hearing aids. 如果我戴上助听器, 我能在会议或演讲中听懂发言人/演讲者的话。	**0.813**	0.200	0.077	0.172	0.736
21. I could understand a one-on-one conversation in a noisy place if I wore hearing aids. 如果我戴上助听器, 我可以在嘈杂的地方听懂–对–的谈话。	**0.783**	0.004	0.142	0.259	0.700
22. I could understand conversation in a small group while in a noisy place if I wore hearing aids. 如果我戴上助听器, 我可以在嘈杂的地方听懂–对多人的谈话。	**0.791**	−0.078	0.006	0.009	0.632
23. I could understand a public service announcement over the loudspeaker in a public building if I wore hearing aids. 如果我戴上助听器, 在公众场合我能听懂扩音器播放的公共服务信息。	**0.840**	0.069	0.055	0.163	0.740
24. I could understand conversation in a car if I wore hearing aids. 如果我戴上助听器, 在车里, 我能听懂别人说话。	**0.857**	0.050	0.100	0.112	0.760
Variance contribution rate after rotation (%)	24.400	20.282	14.283	12.342	–
Cumulative variance contribution rate after rotation (%)	24.400	44.682	58.965	71.307	–
KMO value	0.861				–
Bartlett spherical value	2895.612				–
p value	0.000				–

KMO: Kaiser-Meyer-Olkin.

The C-MARS-HA was conducted on-site by trained audiologists. The audiologists began by explaining to the participants that the 24 items in the questionnaire were about their ability to engage in certain activities related to hearing aids or their subjective perception of hearing when wearing hearing aids in certain situations. For each item, the participants were instructed to write or state a certain number (0%−100% as mentioned previously) that best represented their current ability or feeling. If the participants had never experienced a certain situation, they were asked to anticipate how well they would accommodate to the situation.

In addition to the 24 items included in the C-MARS-HA, we investigated the overall satisfaction of participants with the use of hearing aids by asking a standard question“您对您的助听器总体满意程度为______%([what is] Your overall satisfaction level with your hearing aids ______%).” reflecting the effectiveness of hearing aid use from different perspectives. The scores range from 0%−100%; 0% indicates complete dissatisfaction, while 100% indicates complete satisfaction.

### Statistical analysis

After the survey was completed, the scores for each item were entered into a computer by two researchers and the data were revised as needed. All data were processed using SPSS Version 26.0. Reliability analysis including Cronbach’ s α, Spearman-Brown, and Guttman split-half coefficients; all had reference values greater than 0.6. Validity analysis included content and structural validity. Content validity was evaluated by peer review, whereas construct validity was analyzed by exploratory and confirmatory factor analyses, in which the Bartlett sphericity test showed a statistically significant difference (p < 0.05).

Descriptive statistical analyses of the total and four-factor scores of the C-MARS-HA and overall satisfaction were conducted. The total score was the average score of the 24 items, and the score for each factor represented the average score of the corresponding items. Smith and West suggested that the self-efficacy score of an effective hearing aid should reach 80%; therefore, the proportion of participants who achieved 80% was calculated [8]. A correlation analysis was conducted to reflect the relationship between the four factors and the relationship between the four factors and the total score. As the total score and the scores of the four factors did not conform to a normal distribution, Spearman’s correlation was used for analysis. Statistical significance for the correlation was considered at p < 0.05. For correlation analysis, the correlation was considered moderate or above when the absolute value of the correlation coefficient reached 0.5. Coefficients less than 0.5 were considered as low to moderate. The overall satisfaction score for the hearing aids was obtained from the corresponding questions.

## Results

### General information

**Fig 2:**
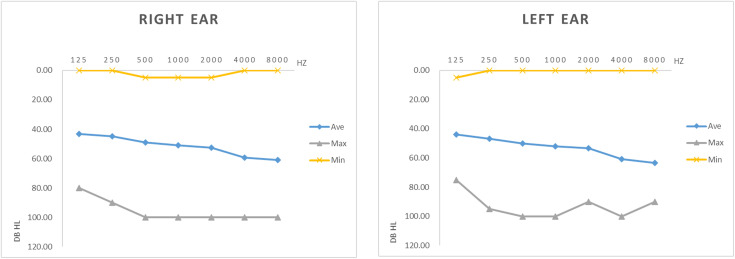
The hearing threshold of the pure tone test. Ave, average value; Max, maximum value; Min, minimum value.

This study included 134 participants aged 20–98, including 57 males and 77 females. 41 participants wore unilateral hearing aids, and 93 participants wore bilateral hearing aids. The types of hearing aids included BTE (Behind the ear), RIE (Receiver in ear), ITE (In the ear), ITC (In the canal), CIC (Complete in canal). The general information of the participants is shown in Table 2, and the degree of hearing loss is shown in Fig 2.

**Table 2:** General information.

	Sex	Number	Age	HA side	HA style	
	Male	55 (42.5%)	68.81 ± 18.11	Right 23(17.2%)	BTE	Right 2(1.72%)
						Left 1(0.91%)
	Female	77(57.5%)	61.79 ± 18.76	Left 18(13.4%)	RIE	Right 50(43.10%)
						Left 47(42.34%)
				Bilateral 93(69.4%)	ITE	Right 28(24.14%)
						Left 27(24.32%)
					ITC	Right 27(23.28%)
						Left 25(22.52%)
					CIC	Right 9(7.76%)
						Left 11(9.91%)
Total		134(100%)	64.77 ± 18.74	134(100%)		Right 116(100%)
						Left 111(100%)

HA, Hearing aids; BTE, Behind the ear; RIE, Receiver in ear; ITE, In the ear; ITC, In the canal; CIC, Complete in canal

### Reliability

#### Internal consistency.

The overall Cronbach’ s α of the C-MARS-HA was 0.914, and the Cronbach’ s α of all four factors was greater than 0.6 (Table 3), indicating that the C-MARS-HA has good internal consistency.

**Table 3:** Results of reliability analysis and confirmatory factor analysis of the C-MARS-HA.

Factors	Convergent validity		Discriminant validity				reliability analysis		
	AVE	CR	Aided listening	Basic handling	Adjustment	Advanced handling	Cronbach’s α	Spearman-Brown	Guttman split-half
Aided listening	0.598	0.930	**0.774** ^**a**^				0.929	0.897	0.896
Basic handling	0.639	0.922	0.234^b^	**0.799** ^**a**^			0.882	0.851	0.842
Adjustment	0.928	0.975	0.422^b^	0.401^b^	**0.964** ^**a**^		0.972	0.978	0.864
Advanced handling	0.565	0.865	0.352^b^	0.438^b^	0.458^b^	**0.752** ^**a**^	0.855	0.780	0.764
Total score							0.914	0.625	0.621

^a^In discriminant validity, numbers represent the AVE square root value; ^b^numbers represent the correlation coefficient.

AVE, average variance extraction; CR, combined reliability; C-MARS-HA, Chinese version of the Measure of Audiologic Rehabilitation Self-Efficacy for Hearing Aids.

#### Split-half reliability.

Split-half reliability analysis was mainly used to complement other reliability indicators such as Cronbach’s α, and to comprehensively evaluate the internal consistency of the questionnaire. The C-MARS-HA was divided into two parts based on odd and even item numbers. The split-half reliability analysis included Spearman Brown and Guttman split-half coefficients for the scores of the total, and each factor was calculated, all of which were greater than 0.6 (Table 3), indicating that the C-MARS-HA has good split-half reliability.

### Validity

#### Content validity.

For content validity, questionnaire items were rigorously tested and repeatedly reviewed. During the introductory process, several experts from the Department of Otolaryngology strictly followed the standard process of the adjusted version of the Brislin translation model. The comprehensibility, logicality, and cultural adaptability of the Chinese version of the questionnaire were tested by a small sample of 30 participants. And familiarity check was conducted by researchers and 6 experts.

Some items in the questionnaire translation process had been revised. In item 8 (I can stop a hearing aid from squealing), the word “squealing” had been translated from “尖叫声” to “啸叫声” to help participants better understand that it referred to the whistling caused by factors such as improper wearing of the hearing aid. In item17 (I could understand conversation in a small group in a quiet place if I wore hearing aids), the translation of “conversation in a small group” had been translated from “小组间的对话” to “–对多人的谈话” to help participants better understand that this referred to conversations between one to many people. This adjustment formed a good progression with the translation of “a one-on-one conversation (–对–的谈话)” in item 16. Similarly, completed the translation adjustment for item 22.

Through the standard process above, ultimately achieved basic consistency between the Chinese and original English versions of the questionnaire in concept, semantics, and context. This confirmed the content validity of the C-MARS-HA.

#### Construct validity.

The results for construct validity are shown in Table 1. First, exploratory factor analysis was conducted on 134 samples, with a Kaiser-Meyer-Olkin (KMO) value of 0.861 and a Bartlett spherical value of 2895.612 (p < 0.01), indicating that the questionnaire results are very suitable for extracting information and reflect the good validity of the C-MARS-HA. Therefore, the questionnaire is suitable for factor analysis.

Next, principal component analysis (PCA) was used to conduct an exploratory factor analysis of the C-MARS-HA. In this study, based on the Kaiser criterion, 5 principal components greater than 1 were initially selected. Then, combined with the scree plot of Scree Text, cumulative variance contribution rate, and the number of factors in the original English questionnaire, it was observed that under the condition of 4 factor numbers, the cumulative variance contribution rate reached 71.307%, indicating that the selected principal components could already represent most of the information in the original data well. Therefore, after maximum variance rotation was completed, the final number of factors was determined to be 4. The cumulative variance contribution rate of the common factors was greater than 40%, the load coefficients of the corresponding factors were all greater than 0.4, and the commonality of all items was greater than 0.4. This indicates that the C-MARS-HA has good construct validity, and the corresponding relationship structure between items and factors is consistent with that of the original English version.

Next, confirmatory factor analysis was conducted on the 24 items. Convergent and discriminant validity tests were conducted (Table 3). The results of the convergent validity tests are represented by the average variance extraction value (AVE) and combined reliability value (CR). The AVE values of the four factors were all greater than 0.5, and the corresponding CR values were all greater than 0.7, indicating that the C-MARS-HA has good convergent validity.

The results of the discriminant validity tests are represented by the AVE square root value and the correlation coefficient between the factors. The AVE square root value represents the aggregation of factors and the correlation coefficient between factors represents the correlation relationship. When the AVE square root value of each factor is greater than the maximum correlation coefficient between that factor and other factors, it indicated that the questionnaire has good discriminant validity. The results of this analysis showed that the minimum AVE square root value corresponding to the four factors was 0.752, which was greater than the maximum value of the correlation coefficient between the factors (0.458), indicating that the C-MARS-HA has good discriminant validity.

### The self-efficacy results of the C-MARS-HA

Descriptive statistical analysis was conducted on the total score and four factors of the C-MARS-HA and overall satisfaction with hearing aids among the 134 participants (Table 4). The average scores for basic handling and adjustment were greater than 80% (92.60% and 81.69%, respectively), and the proportions of users with scores reaching 80% for basic handling and adjustment were greater than 70% (91.04% and 70.90%, respectively), indicating that most users had good self-efficacy in basic handling and adjustment. However, the average scores for aided listening, advanced handling, and the total score were less than 80% (74.76%, 64.54% and 78.70%, respectively). A total of 58.96% of the participants reached the suggested level for the total score, whereas less than 50% of the participants reached the suggested level in aided listening and advanced handling (49.25% and 42.54%, respectively), indicating that most participants had poor self-efficacy in aided listening and advanced handling. The average overall satisfaction score was 81.14%, with 65.67% of the participants reaching 80%, indicating that more than half of the users were satisfied with the effectiveness of the hearing aids.

**Table 4:** Results of descriptive statistics of the C-MARS-HA survey.

	Total score	Aided listening	Basic handling	Adjustment	Advanced handling	Overall satisfaction
Average value (%)	78.70	74.76	92.60	81.69	64.54	81.14
Median value (%)	81.88	78.89	97.14	90.00	70.00	80.00
Standard deviation	15.10	20.96	12.18	23.43	28.84	16.88
Minimum value (%)	37.08	11.11	15.71	0	0	20.00
Maximum value (%)	100.00	100.00	100.00	100.00	100.00	100.00
The proportion of scores ≥80% (%)	58.96	49.25	91.04	70.90	42.54	65.67

C-MARS-HA, Chinese version of the Measure of Audiologic Rehabilitation Self-Efficacy for Hearing Aids.

Spearman’s correlation analysis was conducted on the total score, scores of the four factors of the C-MARS-HA, and overall satisfaction score (Table 5). The correlations between the total score and the four factors, as well as between the four factors, were significant (p < 0.001). The correlations between aided listening and basic and advanced handling were the lowest (0.314 and 0.333, respectively), whereas the total score had the highest correlations with aided listening and advanced handling (0.774 and 0.783, respectively). The correlations between the total score, scores of the four factors, and the overall satisfaction score were significant (p < 0.001). These results indicate a significant positive correlation between the total score, four factors, and overall satisfaction with the C-MARS-HA. Moreover, the strength of the correlation varies.

**Table 5:** Results of Spearman correlation analysis between the C-MARS-HA and overall satisfaction.

	Total score	Aided listening	Basic handling	Adjustment	Advanced handling	Overall satisfaction
**Total score**	1	0.774***	0.613***	0.681***	0.783***	0.474***
**Aided listening**		1	0.314***	0.404***	0.333***	0.454***
**Basic handling**			1	0.453***	0.492***	0.322***
**Adjustment**				1	0.486***	0.445***
**Advanced handling**					1	0.315***
**Overall satisfaction**						1

***: indicates p < 0.001; C-MARS-HA, Chinese version of the Measure of Audiologic Rehabilitation Self-Efficacy for Hearing Aids.

## Discussion

### The reliability and validity of the C-MARS-HA

West and Smith developed the MARS-HA and conducted a reliability and validity analysis of the survey results of new and experienced users of hearing aids (who were distinguished by whether they had used hearing aids for more than 6 months). The results showed that the MARS-HA has good reliability and validity [7].

For reliability verification in this study, the internal consistency and split-half reliability of the overall and four factors were analyzed (Table 3). The internal consistency was represented by Cronbach’s α. The overall and four factors of Cronbach’s α were all greater than 0.8 (the minimum value was 0.855), indicating that the C-MARS-HA has high reliability. The split-half reliability was represented by the Spearman-Brown and Guttman split-half coefficients. These two coefficients for the overall and four factors were both greater than 0.6 (the minimum value was 0.621), further confirming that the reliability of the C-MARS-HA meets statistical requirements.

For validity verification in this study, this study analyzed the content and construct validity of the Chinese version of the MARS-HA. First, after repeated experimentation and reviews strictly following the Brislin translation model for cross-cultural translation, file testing and familiarity check, the translated questionnaire was found to have good content validity (Fig 1). The MARS-HA also has Spanish and French versions, both of which used similar cross-cultural translation methods and have good validity [18,19].

Subsequently, through exploratory factor analysis (Table 1), four common factors that met the statistical requirements were extracted from the 24 items of the C-MARS-HA: Factor 1 (aided listening, corresponding to items 16–24), Factor 2 (basic handling, corresponding to items 1–5,7,10), Factor 3 (adjustment, corresponding to items 13–15), and Factor 4 (advanced handling, corresponding to items 6,8,9,11,12), which were consistent with the factors of the original questionnaire. After rotation, the cumulative variance contribution rate of the common factors was greater than 40% (reached 70.307%), and the load coefficients of the corresponding factors were all greater than 0.4 (the minimum value was 0.466). The commonality of all items was greater than 0.4 (the minimum value was 0.424), indicating that the C-MARS-HA has good structural validity.

A confirmatory factor analysis was performed for further testing (Table 3). The AVE values of all four factors were greater than 0.5 (the minimum value was 0.565), and the CR values were greater than 0.7 (the minimum value was 0.865), indicating that the C-MARS-HA has good convergent validity. The minimum square root value (0.752) of the AVE corresponding to the four factors was greater than the maximum value (0.458) of the correlation coefficient between the factors, indicating that the C-MARS-HA has good discriminant validity.

Based on the reliability and validity analysis of C-MARS-HA mentioned above, the questionnaire has good reliability and validity.

### The self-efficacy results of the C-MARS-HA

The results of the C-MARS-HA survey targeting 134 participants showed self-efficacy of those participants (Table 4). The hearing aid users scored 80% of the recommended level for basic handling (reached 92.60%) and adjustment (reached 81.69%), and that the proportion of users who reached this level exceeded 70% (reached 70.90% and 91.04%). The scores for aided listening and advanced handling were relatively low (reached 74.76% and 64.54%). These results are consistent with those of other studies of the MARS-HA conducted in multiple countries and regions [7,14–19]. Approximately 66% of the users achieved a good level of overall satisfaction with hearing aids.

The score for basic handling was relatively high. This may have occurred because skills such as battery replacement are frequently performed by users. Hearing aid users usually receive training during their initial fitting; therefore, one would expect that they would be more proficient in these skills and have a higher level of confidence [15]. The adjustment score was also better, which may be because all the participants included in this study were experienced hearing aid users. Audiologists adjusted the hearing aids based on the user’ s hearing levels, and regular follow-up visits were conducted to adjust the hearing aid settings according to the usage. Any problems encountered by the users were promptly addressed.

The low self-efficacy in aided hearing may have been due to the following. First, all participants included in this study were experienced hearing aid users. After using hearing aids for a certain period, experienced users are aware that hearing aids cannot solve all hearing problems [7]. Second, most participants were of advanced ages, and hearing impairment may worsen with age, leading to more obvious problems. Low self-efficacy in advanced handling is common, indicating that troubleshooting and similar operations are challenging for hearing aid users [7,14–19]. In this survey, most users believed that advanced handling requires professional personnel, and that they do not have the ability to perform tasks involved with advanced handling of the hearing aids. Therefore, upon encountering these types of problems, the hearing aid users will seek the help of professionals.

The scores of the total and four factors of the C-MARS-HA had significant positive correlations with each other (Table 5), further verifying the good internal consistency of the C-MARS-HA. These results are consistent with the results of the Spanish version of the questionnaire survey [18]. The difference in correlation strength also reflects the need for an overall evaluation of self-efficacy with an emphasis on different factors. The C-MARS-HA scores also had a significant positive correlation with overall satisfaction, with differences found in the correlations between each item. The correlation between overall satisfaction and aided listening or adjustment was relatively high compared with those of basic and advanced handling, indicating a higher correlation between user satisfaction and auditory perception.

Our survey revealed an average total score for self-efficacy of hearing aid users of 78.70%, and an average score for overall satisfaction of 81.14%, indicating that the self-efficacy of the hearing aid users could be improved. Providing simple and understandable written materials and video demonstrations and engaging the significant others of users to participate in the daily management and rehabilitation training of hearing aids may lead to further improvements in self-efficacy [8,14,21].

### Limitations

In this study, we invited experienced hearing aid users and conducted a questionnaire survey on them. We focused on using MARS-HA to assess participants’ subjective feelings and future research can further compare the correlation between subjective and objective data. Due to the uncertainty of participants follow-up and the limitation of research time, there are shortcomings in this study in terms of test-retest reliability, number of participants, long-term performance of self-efficacy, and analysis of factors affecting self-efficacy. In future research, we will try our best to arrange the research plan reasonably, overcome existing difficulties, and strive to further improve the study and enhance the quality of the research.

## Conclusion

In summary, our results revealed that the C-MARS-HA has good reliability and validity. This tool is suitable for clinical evaluation of the confidence level of users in their abilities to listen with, handle, and adjust hearing aids, reflecting the benefits and difficulties of hearing aid use. The survey results showed that hearing aid users from China have good self-efficacy in basic handling and adjustment but poor self-efficacy in aided listening and advanced handling. And hearing aid self-efficacy is associated with overall satisfaction with hearing aids. The information derived from this questionnaire will assist audiologists in planning and managing the care of individuals using hearing aids.

## Supporting information

S1 Annex The Chinese version of audiologic rehabilitation self-efficacy for hearing aids (C-MARS-HA). (DOCX)

S2 Annex English version of C-MARS-HA. (DOCX)

S1 Data Data used in all analyses. (XLSX)
