# Supplementary material for: Translation of Chinese version of the measure of audiologic rehabilitation self-efficacy for hearing aids and the self-efficacy among hearing aid users in China: Application of the questionnaire
Source: PLoS One. 2025 Aug 20;20(8):e0330163. doi: 10.1371/journal.pone.0330163 (PMC12367113; doi:10.1371/journal.pone.0330163)

**自我效能问卷MARS-HA-中文版**

**姓名： 性别： 出生年/月/日： / / /**

**填表日期年/月/日： / / /**

**请提供以下您的助听器使用情况：**

1. 听力下降多久选择使用助听器： 月
2. 目前助听器佩戴侧别：

| ○左侧 | ○右侧 | ○双侧 |
| --- | --- | --- |

1.
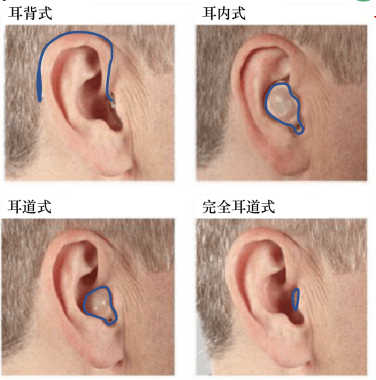
目前助听器佩戴时间： 月
2. 一生（包括目前）助听器使用经验： 月
3. 每天助听器使用时间： 小时/天；过去的两周，佩戴了 ________天？
4. 佩戴助听器款式：

| ○耳背式 | ○耳内式 | ○耳道式 | ○完全耳道式 |
| --- | --- | --- | --- |

1. 您对您的助听器总体满意程度为______%。（ 0%为完全不满意，100%为非常满意）

**下列内容填写说明：**

以下问题是关于您使用助听器时做某些活动的能力或在某些情形下的听力主观感受。对于每项题目，请圈画出最代表您**目前**感受的数字，下方列出每个数字所代表的意思：


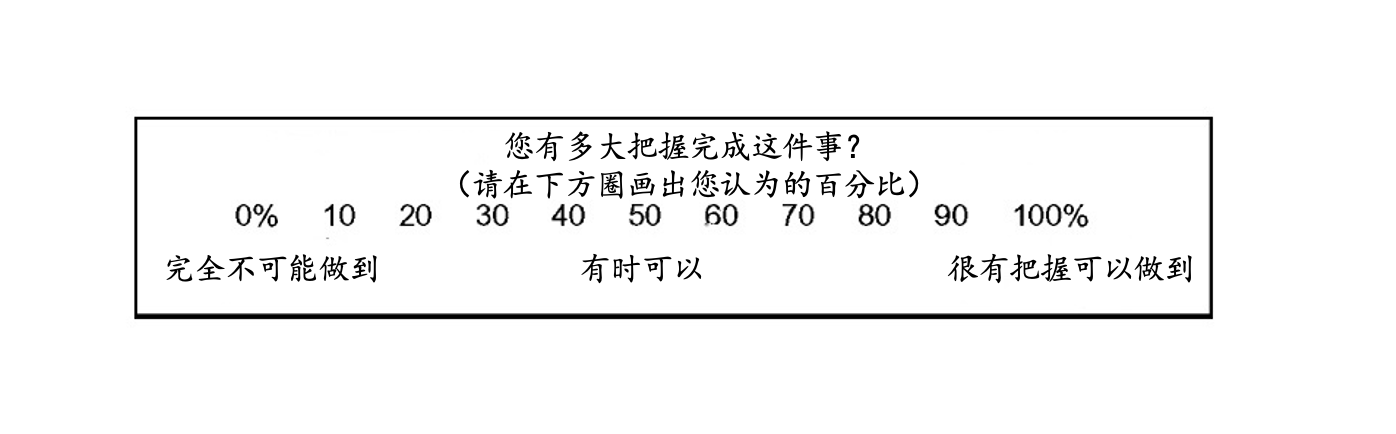


请记得您的回答将会表达出您对目前使用助听器的整体评价。如果你从未经历过这些情况，那么请尽您最大的努力去猜测您能做得多好。

1. 我可以很容易地把电池装到助听器里。


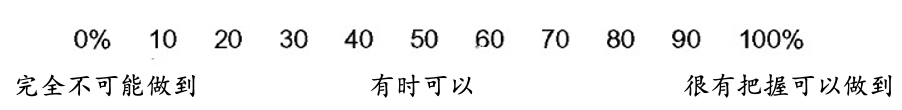


1. 我可以很容易地把电池从助听器里取出。


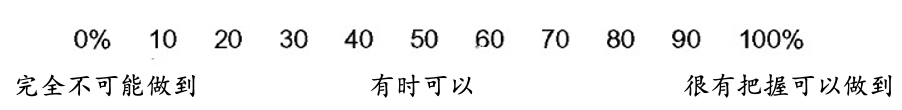


1. 我能分辨出助听器是左耳的还是右耳的。


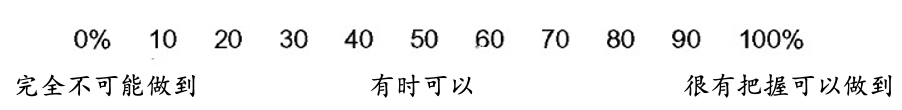


1. 我可以准确地把助听器放入我的耳朵。


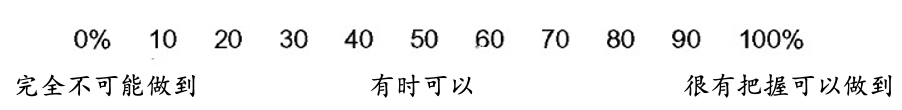


1. 我可以很容易地把助听器从耳朵里拿下来。


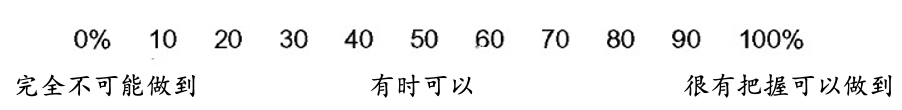


1. 我可以识别特定助听器的不同部件（比如麦克风、电池仓、通气孔等）。


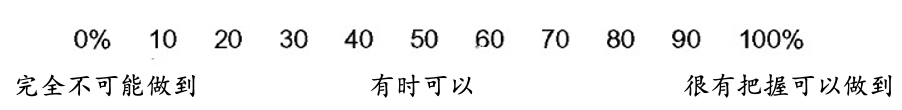


1. 我可以操作助听器上的所有按钮(旋钮、开关、遥控器（如有）等）。


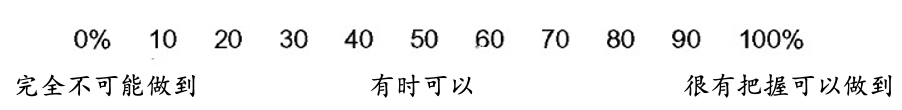


1. 我可以使助听器不发出啸叫声。


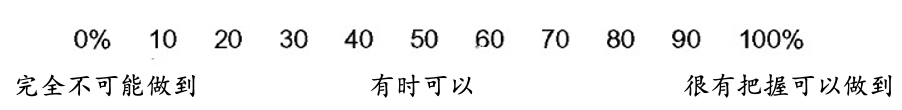


1. 助听器有问题的时候，我可以进行故障排除。


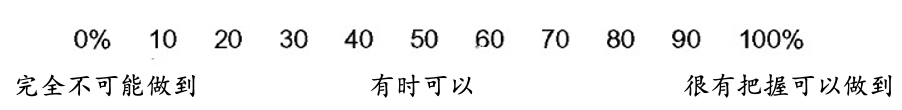


10.我会定期清洁和保养助听器。


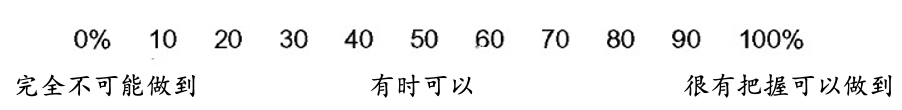


1. 我可以说出助听器的牌子或型号。


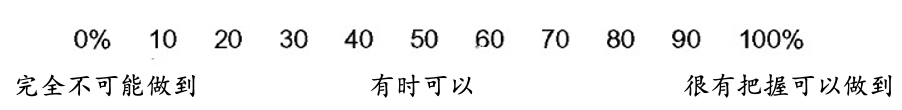


1. 我可以说出助听器所需的电池型号。


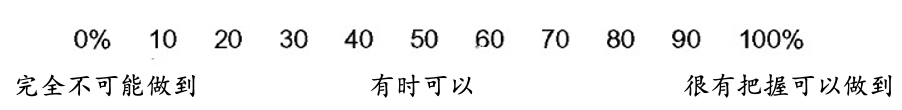


1. 我可以习惯助听器的音质。


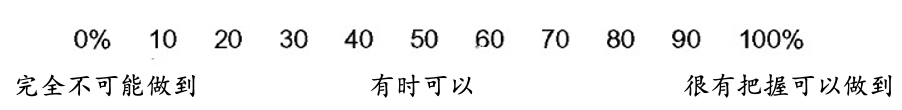


1. 我可以习惯助听器在我耳朵里的感觉。


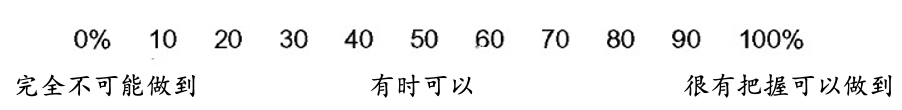


1. 如果我戴上助听器，我可以习惯我自己的声音。


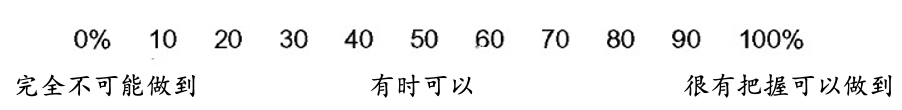


1. 如果我戴上助听器，我可以在安静的地方听懂一对一的谈话。


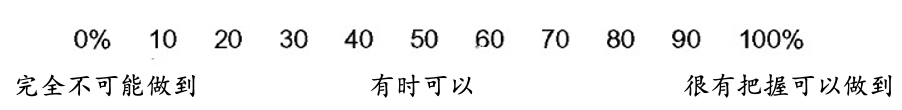


1. 如果我戴上助听器，我可以在安静的地方听懂一对多人的谈话。


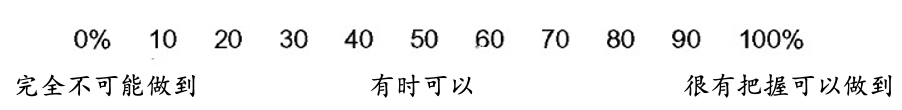


1. 如果我戴上助听器，我能通过电话进行交流。


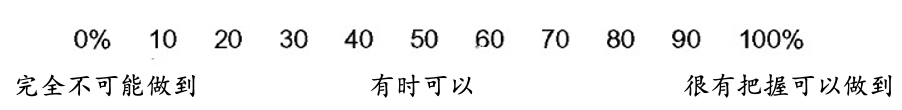


1. 如果我戴上助听器，我能看电视。


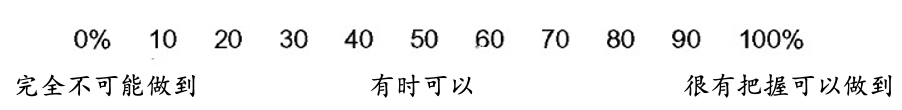


1. 如果我戴上助听器，我能在会议或演讲中听懂发言人/演讲者的话。


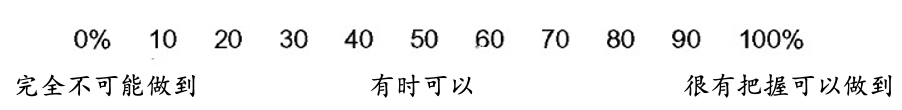


1. 如果我戴上助听器，我可以在嘈杂的地方听懂一对一的谈话。


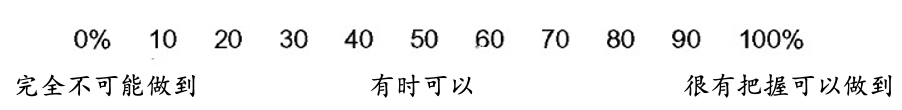


1. 如果我戴上助听器，我可以在嘈杂的地方听懂一对多人的谈话。


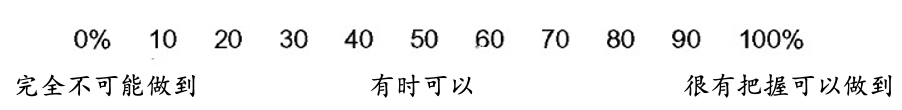


1. 如果我带上助听器，在公众场合我能听懂扩音器播放的公共服务信息。


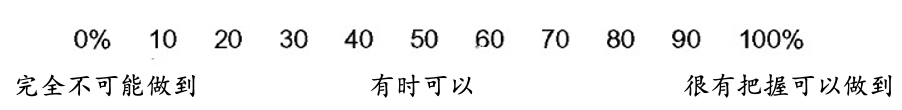


1. 如果我戴上助听器，在车里，我能听懂别人说话。


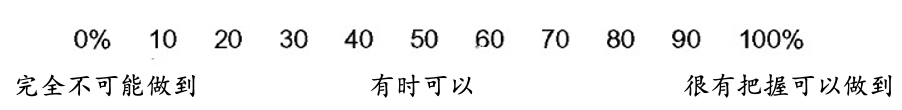

Supplement: S1 Annex — (DOCX) [file pone.0330163.s001.docx]
