# Supplementary material for: Translation of Chinese version of the measure of audiologic rehabilitation self-efficacy for hearing aids and the self-efficacy among hearing aid users in China: Application of the questionnaire
Source: PLoS One. 2025 Aug 20;20(8):e0330163. doi: 10.1371/journal.pone.0330163 (PMC12367113; doi:10.1371/journal.pone.0330163)

**Chinese Version of Measure of Audiologic Rehabilitation Self-Efficacy for Hearing Aids (MARS-HA) Questionnaire**

**Name： Gender： Date of Birth (Y/M/D)： / / /**

**Date (Y/M/D)： / / /**

Please provide the following information regarding your hearing aid use:

1. Duration of Hearing Loss Before Selecting a Hearing Aid: _____ months
2. Current Side of Hearing Aid Usage:

○ Left side ○ Right side ○ Both sides

1.
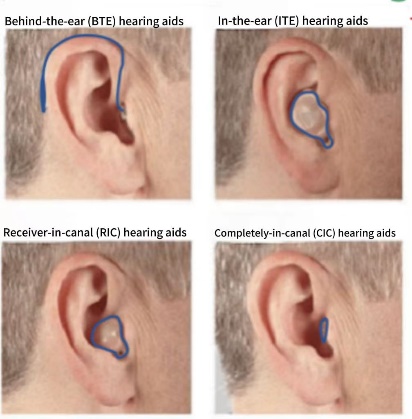
 Current Duration of Hearing Aid Usage: _____ months
2. Lifetime Experience with Hearing Aid (Including Current): _____ months
3. Average Daily Hearing Aid Usage:
   _____ hours/day; Over the past two weeks, how many days have you used it? _____ days
4. Style of Hearing Aid Worn:

○ Behind-the-ear ○ In-the-ear ○ In-the-canal ○ Completely-in-the-canal

1. Overall Satisfaction with Hearing Aid: _____% (0% = Not satisfied at all; 100% = Completely satisfied)

Instructions for Completing the Following Section:

The following questions pertain to your ability to perform certain activities or your subjective hearing experiences in specific situations while using a hearing aid. For each question, please circle the number that best represents your current experience. The meaning of each number is listed below:

How certain are you that you can do this? (circle percentage)

| 0% | 10 | 20 | 30 | 40 | 50 | 60 | 70 | 80 | 90 | 100% |  |
| --- | --- | --- | --- | --- | --- | --- | --- | --- | --- | --- | --- |
| Cannot do Moderately I am certain  this at all certain can do I can do this | | | | | | | | | | | |

Please remember that your responses will reflect your overall assessment of using your hearing aid at present. If you have never encountered these situations, please make your best estimate regarding how well you believe you would manage.

1. I can insert a battery into a hearing aid with ease.


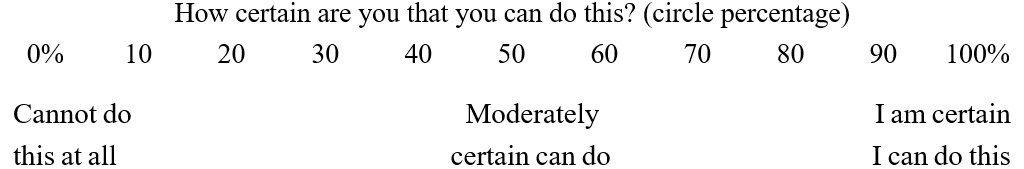


1. I can remove a battery from a hearing aid with ease.


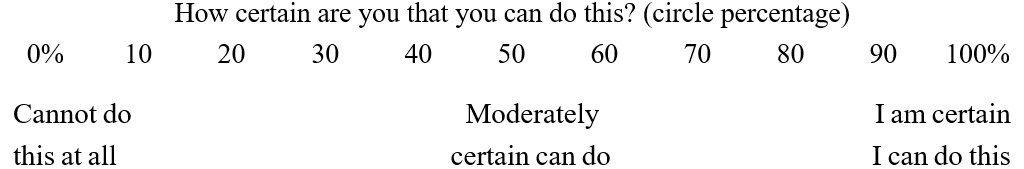


1. I can tell a right hearing aid from a left hearing aid.


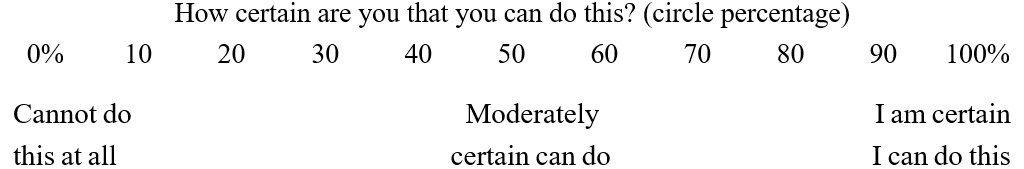


1. I can insert hearing aids into my ears accurately.


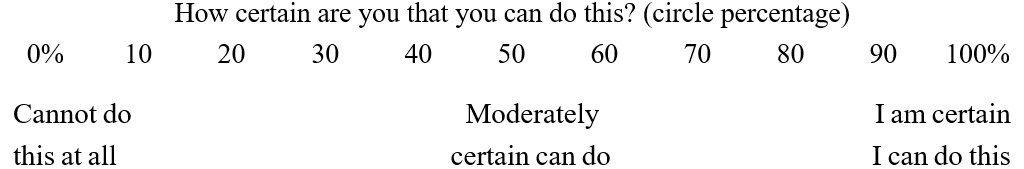


1. I can remove hearing aids from my ears with ease.


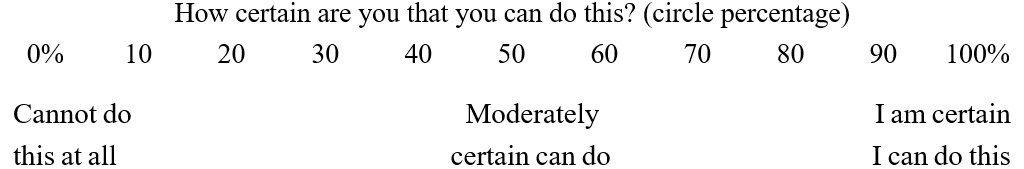


1. I can identify the different components of a particular hearing aid (i.e., microphone, battery door, vent, etc.).


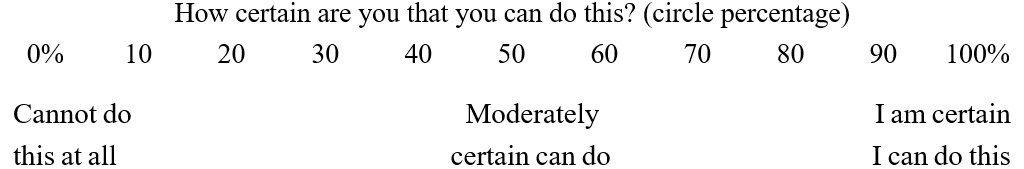


1. I can operate all the controls on a particular hearing aid (knobs, switches, and/or remote control) appropriately.


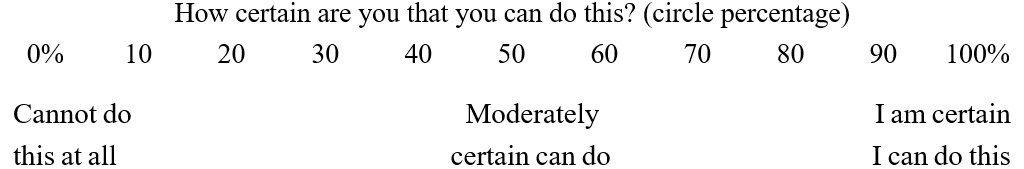


1. I can stop a hearing aid from squealing.


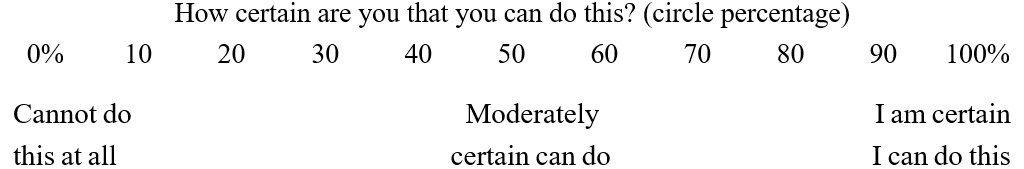


1. I can troubleshoot a hearing aid when it stops working.


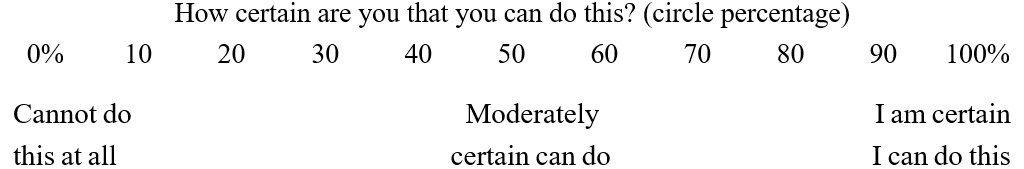


10. I can clean and care for a hearing aid regularly.


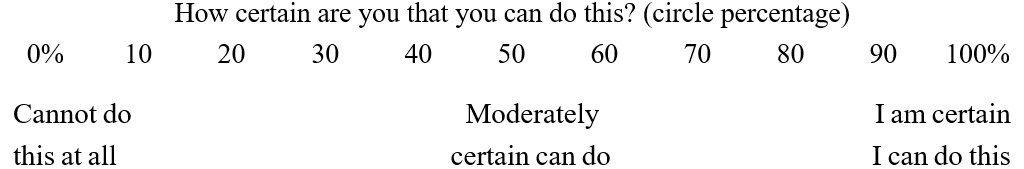


1. I can name the make or model of a particular hearing aid.


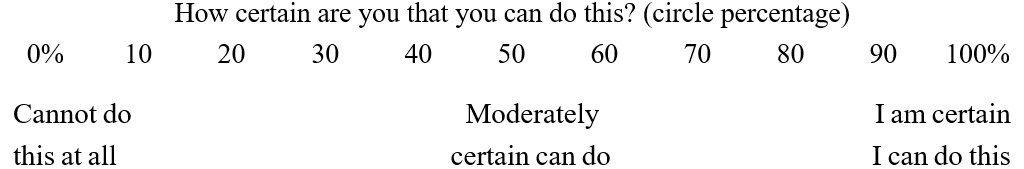


1. I can name the battery size needed for a specific hearing aid.


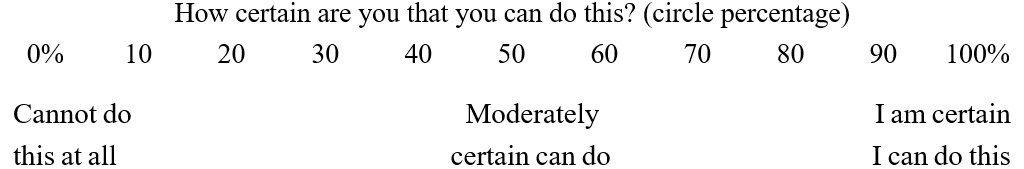


1. I could get used to the sound quality of hearing aids.


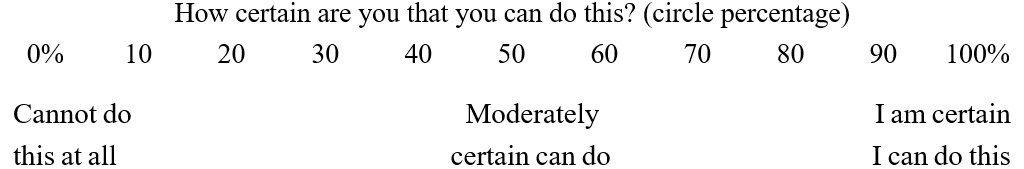


1. I could get used to how a hearing aid feels in my ear.


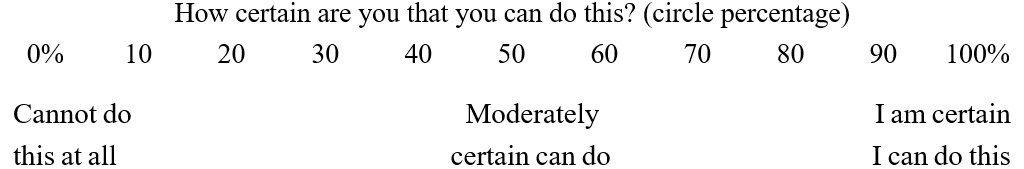


1. I could get used to the sound of my own voice if I wore hearing aids.


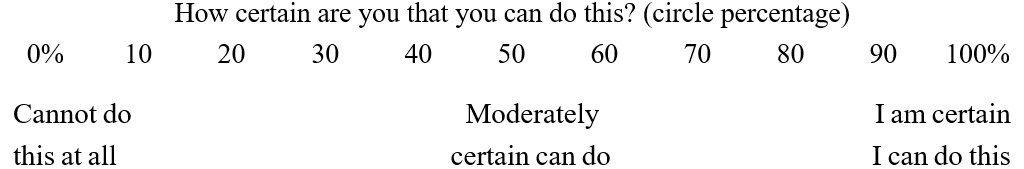


1. I could understand a one-on-one conversation in a quiet place if I wore hearing aids.


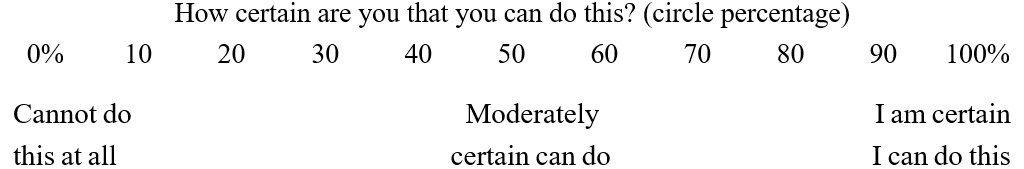


1. I could understand conversation in a small group in a quiet place if I wore hearing aids.


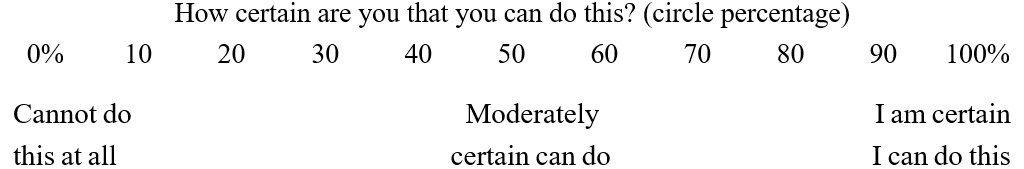


1. I could understand conversation on a standard telephone if I wore hearing aids.


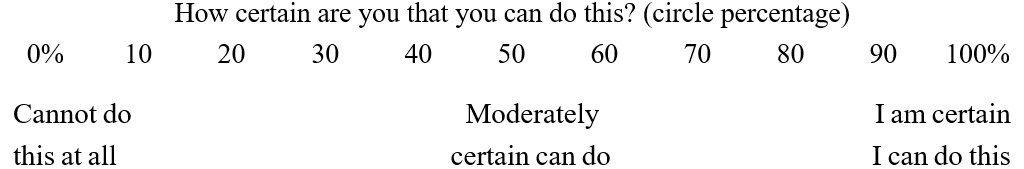


1. I could understand television if I wore hearing aids.


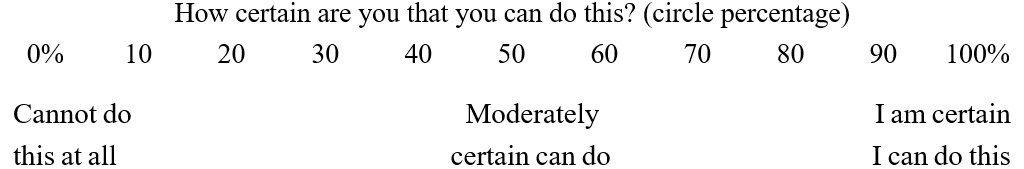


1. I could understand the speaker/lecturer at a meeting or presentation if I wore hearing aids.


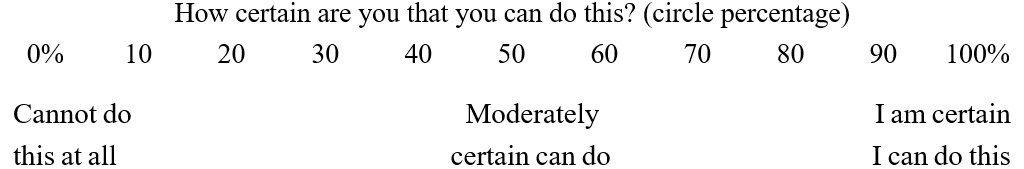


1. I could understand a one-on-one conversation in a noisy place if I wore hearing aids.


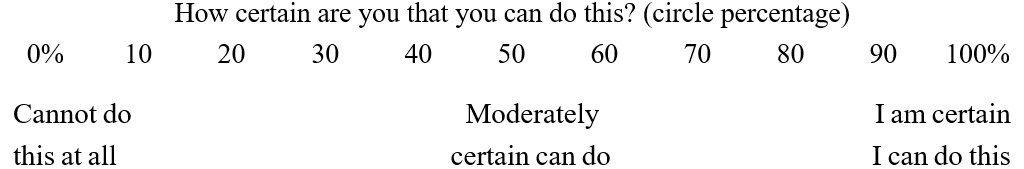


1. I could understand conversation in a small group while in a noisy place if I wore hearing aids.


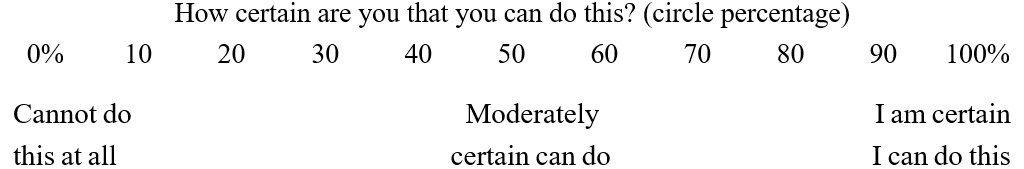


1. I could understand a public service announcement over the loudspeaker in a public building if I wore hearing aids.


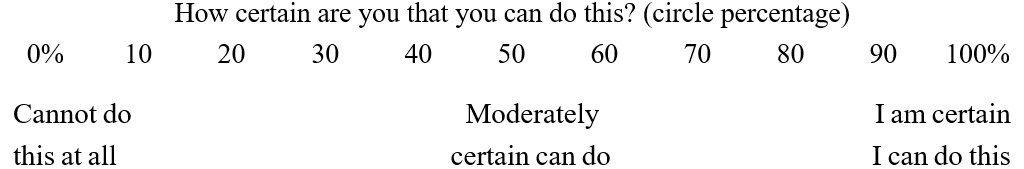


1. I could understand conversation in a car if I wore hearing aids.


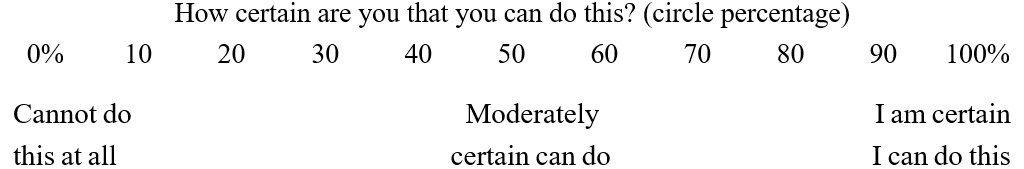

Supplement: S2 Annex — (DOCX) [file pone.0330163.s002.docx]
